# A method for the estimation of a motor unit innervation zone center position evaluated with a computational sEMG model

**DOI:** 10.3389/fnbot.2023.1179224

**Published:** 2023-07-06

**Authors:** Malte Mechtenberg, Axel Schneider

**Affiliations:** Biomechatronics and Embedded Systems Group, Bielefeld University of Applied Sciences and Arts, Bielefeld, Germany

**Keywords:** innervation point, motor endplate, sEMG simulation, concentrated current source, motor unit (MU), conduction velocity (CV), exoskeleton, innervation zone

## Abstract

Motion predictions for limbs can be performed using commonly called Hill-based muscle models. For this type of models, a surface electromyogram (sEMG) of the muscle serves as an input signal for the activation of the muscle model. However, the Hill model needs additional information about the mechanical system state of the muscle (current length, velocity, etc.) for a reliable prediction of the muscle force generation and, hence, the prediction of the joint motion. One feature that contains potential information about the state of the muscle is the position of the center of the innervation zone. This feature can be further extracted from the sEMG. To find the center, a wavelet-based algorithm is proposed that localizes motor unit potentials in the individual channels of a single-column sEMG array and then identifies innervation point candidates. In the final step, these innervation point candidates are clustered in a density-based manner. The center of the largest cluster is the estimated center of the innervation zone. The algorithm has been tested in a simulation. For this purpose, an sEMG simulator was developed and implemented that can compute large motor units (1,000's of muscle fibers) quickly (within seconds on a standard PC).

## 1. Introduction

For the control of exoskeletons and wearables, different strategies are used to adapt the motion of the technical system to the motion of a limb. Often, the forces in the mechanical interfaces between the body and the technical system (braces, straps, etc.) are measured and are used to control the motion of the technical system (for a review on control strategies for upper-limb exoskeletons, see, e.g., Dalla Gasperina et al., 2021). However, since the forces arise at a moment when the technical system should already be responding to the movement, the wearables inevitably lag behind. A movement prediction for the limb would therefore be desirable. The measurements of surface electromyograms (sEMGs) of the muscles involved in the limb movement are suitable for this prediction, since sEMGs can already be measured a few tens of milliseconds before the muscles generate force [electromechanical delay, see, e.g., ~ 106ms for the human knee extensor (Vos et al., 1991) and ~ 53ms for human upper limb muscles (Cavanagh and Komi, 1979; Falk et al., 2009; Cè et al., 2013)]. This time advantage could be exploited for an early movement activation of the technical system to compensate for the mentioned lag. An sEMG-based prediction of a limb movement requires modeling of the muscles (Zajac, 1989; Buchanan et al., 2004; Grimmelsmann et al., 2023), tendons (Mechtenberg et al., 2022), and other biomechanical components of the joint involved. Successful application of a muscle-tendon model requires precise knowledge of the mechanical muscle parameters, muscle length and contraction speed as well as the distribution of the total length of the muscle-tendon complex between its two subcomponents, namely muscle and tendon. These are difficult to determine and track *in vivo* (Grimmelsmann et al., 2023).

This is where the work at hand becomes relevant. An sEMG is normally only used as an activation input for a classical Hill-based muscle model (Zajac, 1989; Grimmelsmann et al., 2023). The actual muscle length and velocity as additional inputs of the modeled muscle are approximated from an estimated initial length and the measured joint motion. In this study, a method to track the center position of the innervation zone is proposed. This method could be used in the future to track the center positions of the innervation zone of a muscle's motor units over time, which provide information about the mechanical contraction motion of the muscle itself and could therefore be used to improve the estimation of the muscle length online, which in turn might improve the prediction of limb motion, e.g., for exoskeleton and wearable control systems.

**Figure 1:**
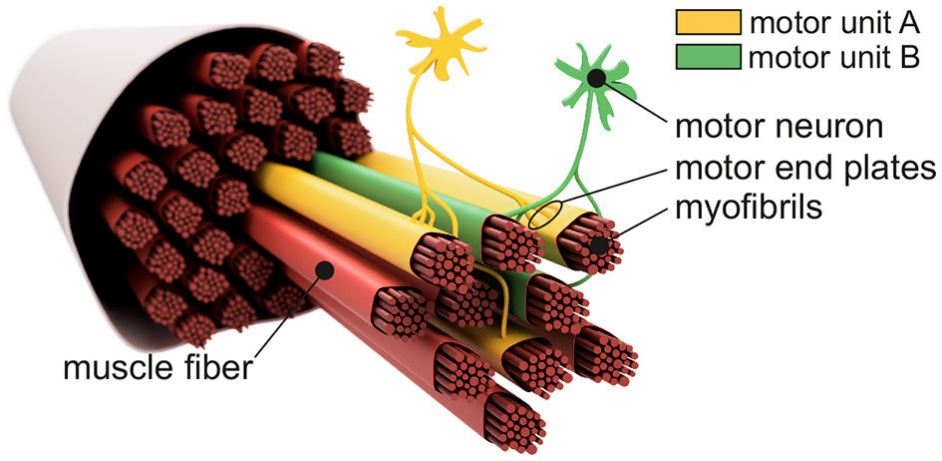
Sketch-like representation of a muscle fiber bundle. The muscle fibers are differentiated by color to indicate their affiliation to different motor units. Highlighted are two motor neurons which, together with their associated (i.e., controlled) muscle fibers, form motor unit A (yellow) and motor unit B (green). Motor end plates indicate individual innervation points. Since the innervation points of a motor unit are spatially distributed over the associated muscle fibers, one can also consider an innervation zone.

**Figure 2:**
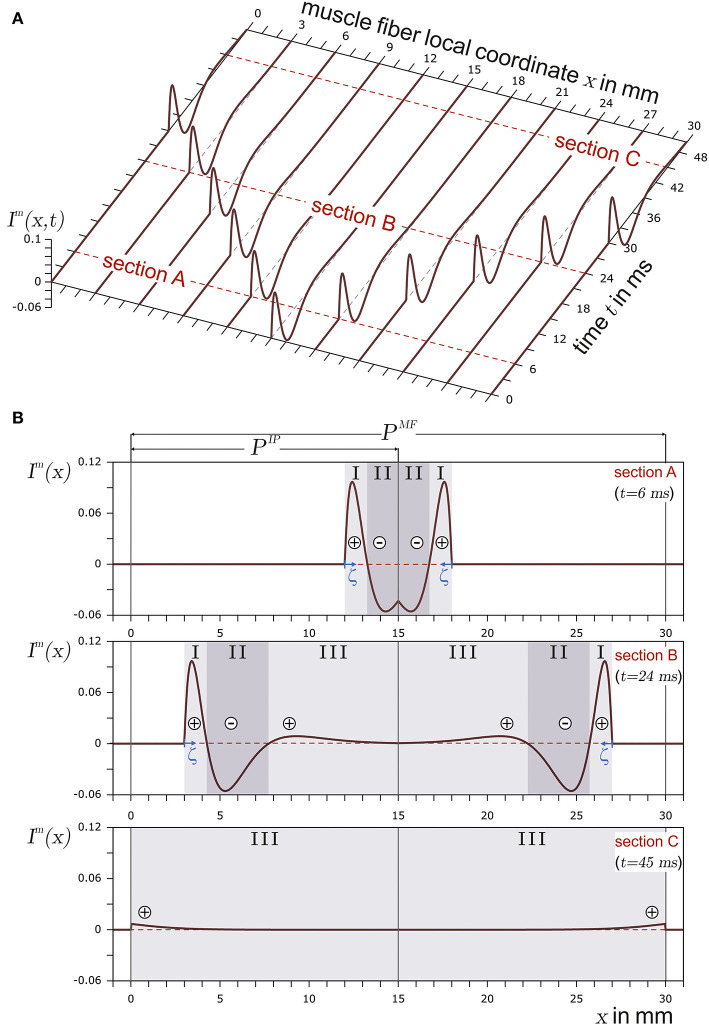
In **(A)**, the current source density of a finite muscle fiber is shown over the muscle fiber local coordinate *x* and time *t*. The muscle fiber parameters are depicted in Figure 3. In **(B)**, time sections are shown. The current source emerges at the innervation point (*t* = 6ms, section A), fully develops at *t* = 24ms in section B, and travels along the muscle fiber. Finally, the current source is almost extinct at the muscle fiber ends (*t* = 45ms) in section C. The center of each concentrated current source/sink is indicated by either ⊕ for the concentrated current source or by ⊖ for the concentrated current sink. The origin of the local coordinate system ζ of current source is indicated in sections A and B. In the case of section C, the local coordinate system of the current source is outside the plot and therefore not displayed.

Figure 1 shows the basic structure of a motor unit consisting of a motor neuron (located in the spinal cord) and the muscle fibers innervated by the motor neuron. The figure shows two arbitrary motor units (A in yellow and B in green). Individual innervation points (motor end plates) of a motor neuron distribute themselves over larger areas (Masuda and Sadoyama, 1991; Amirali et al., 2007) and thus form innervation zones. When all the muscle fibers belonging to a motor unit (one color) are activated by their respective motor neuron, their myoelectric potentials run quasi-simultaneously from the innervation points along the muscle fibers in both directions until they reach the tendon tissue and disappear. This is shown schematically in Figure 2A for a signal starting from a single innervation point. Here, the respective time courses of trans-membrane current densities (or current source densities, which are the basis for myoelectric potentials) are shown at 11 positions along the muscle fiber. It is illustrated how a signal originates from the center and travels in both directions, i.e., it becomes visible at the other positions at a later time. The local distribution in three sections is shown in Figure 2B. On the surface of the skin above the muscle, these potentials appear as sum potentials moving across the muscle.

Various studies have already been presented for the identification of the center position of the innervation zone of those sum potentials that belong to the same motor unit. Mesin et al. (2009) presented an automatic method for the estimation of the position of the innervation zone based on a linear electrode array. The position resolution of the innervation zone is bound to the electrode positions; therefore, if an innervation zone was located between electrodes, the algorithm by Mesin et al. (2009) would assign the innervation zone position to the nearest electrode. Their algorithm can identify multiple innervation zone positions within the same time window. Marateb et al. (2016) also introduced an automatic method for the estimation of the position of the innervation zone based on a linear electrode array. In the case of their algorithm, a single differential electrode array recording is interpreted as an image which is then segmented using the Graph-Cut image processing algorithm. After pruning, motor unit potential regions are identified, and lines are fitted into these regions. The fitted lines are then used to determine the action potential conduction velocity and innervation zone center. The algorithm can predict the position of the innervation zone between electrodes but its accuracy is subject to inter electrode distance. Beck et al. (2012) compared three algorithms which were based on either cross-correlation, the detection of the lowest amplitude channel, or the detection of the highest mean frequency. Of these three, Beck et al. (2012) found that the cross-correlation-based algorithm performed the best.

This study presents a computationally efficient algorithm for fast estimation of the center position of the innervation zone based on wavelets (see Section 2.3). Only two properties are needed to parameterize the algorithm (robustness vs. accuracy, see Section 3.2). Apart from this, the algorithm runs automatically. Furthermore, a myoelectric simulation framework that is also computationally efficient is being introduced to test such algorithms (see Section 2.1 for the algorithm and Section 2.2 for the experimental setup). This framework is capable of determining surface sum potential curves (virtual sEMGs) based on myoelectric signal simulations of hundreds of muscle fibers on a large scale and almost in real time (details on the performance are given in Section 3.1).

The myoelectric simulation framework should be able to simulate motor unit potentials based on a large number of muscle fibers with configurable lengths, innervation point positions, and conduction velocities. Here, the consideration of the signal generation at the innervation point (source function) and the end of the signal at the myotendinous junction (end effect) plays a crucial role. The newly introduced simulator uses concentrated current sources that arise successively from the innervation point, whose strength initially increases to full strength in the vicinity of the innervation point. After their traveling across the fiber, their strength decreases near the myotendinous junction until they finally disappear. In terms of complexity, the new simulator places itself in between models that use a continuous source function (Farina et al., 2004; Petersen and Rostalski, 2019) (more computationally expensive) and dipole-based models that always treat the concentrated dipole current sources in pairs (Stegeman et al., 2000) and are thus further from the continuous description of the current source. Another goal was to design a muscle-independent simulator, since the method presented here for estimating the position of the center of the motor unit innervation zone is not intended to be adapted to a specific muscle. In contrast to the most accurate mapping of a muscle with its specific physiological environment (e.g., bones, blood vessels, and tissue layers), as proposed e.g., by Zhang et al. (2017), the simulator introduced here places the motor unit in a purely resistive, homogeneous, and isotropic medium.

The actual test of the algorithm using the myoelectric simulation framework is carried out in Section 3.2. Here, the simulated sEMG signals are subject to varying levels of noise, and the recognition performance of the algorithm is assessed.

## 2. Methods

For reasons of clarity, the method section begins with a description of the myoelectric simulation framework. An implementation of the presented myoelectric simulation framework is published on GitHub (Mechtenberg, 2023). The next step describes the parameterization of the simulator for an experiment that serves as the basis for testing the algorithm for recognizing the center position of the innervation zone. The algorithm for estimating the center position of the innervation zone is described in the last step.

### 2.1. Myoelectric simulation framework–sEMG simulation

The muscle fiber is a complex single-cell structure with multiple *nuclei* (McCuller et al., 2022). Its membrane has embedded proteins that allow passive and active ion fluxes through the membrane (current source). Upon excitation, these membrane proteins allow for an active ion flux and thus generate a myoelectric action potential, which, in turn, excites the neighboring proteins. Due to a refractory period after excitation, during which the respective active protein cannot be excited, myoelectric action potentials travel one way along the muscle fiber (moving sites of ion flux). The resulting changes in the electrical properties of the muscle fiber can therefore be represented as a traveling current source in space with finite dimensions (Roberto Merletti, 2004). The initial excitation of the muscle fiber membrane occurs at the motor end plate. This is the place where the muscle fiber is innervated, i.e., connected by the axon of a motor neuron. In vertebrates, a single muscle fiber is always innervated by only one motor neuron. This motor neuron in turn connects to multiple muscle fibers and excites all connected muscle fibers at the same time. The motor neuron and its connected muscle fibers are called the motor unit. Motor units can be categorized into three types (S = slow, FR = fast fatigue resistant, and FF = fast fatigable) based on the metabolism of the muscle fiber and properties of force generation (Pette and Staron, 2000; Heckman and Enoka, 2004). Different types of motor units are found to have different physiological parameters such as the amount of muscle fibers per motor unit, the velocity of the traveling myoelectric action potential (conduction velocity), the rise time and duration of the force twitch, and time to fatigue (Burke et al., 1973). This study is mainly focused on the electrical potentials resulting from motor unit excitation. Therefore, relevant motor unit parameters are the number of muscle fibers in a motor unit and the conduction velocity along a single muscle fiber. For the modeling of the generated electrical field of all motor unit myoelectric action potentials, a current source model for each myoeletric action potential consisting of concentrated current sources is introduced (Section 2.1.1). In addition, this also includes a description of the resulting potential field in quasi-static conditions (Section 2.1.2) and the relevant physiological parameters (e.g. the two myotendinous junction regions, the innervation zone region, the number of muscle fibers in a motor unit, and the conduction velocity of the myoelectric action potentials) of a motor unit (Section 2.1.3).

#### 2.1.1. Concentrated current source model of a finite muscle fiber

A myoelectric action potential is a perturbation of the potential difference across the active membrane (resting potential). As described earlier, this perturbation is caused by ion channels that actively and passively transport electrical charges (ion flux) and therefore act as current sources, which in general are called *myoelectric current sources*. The myoelectric current source can be described by measurement data, an approximation function, or by a set of differential equations which model the dynamic behavior of the ion channels. If the trans-membrane current is neither measured directly nor taken from a system model of the membrane components, then it can be derived by fitting a mathematical function to the shape of a membrane potential. The easiest and most intuitive model in the latter context is the *core conductor model*, which has its origin in *cable theory* (Taylor, 1963). Here, an approximated function of the trans-membrane potential *V*^*m*^(ζ) is utilized, from which the trans-membrane current *i*^*m*^(ζ) is derived. The function in Equation 1 is continuous and is therefore widely used to model the trans-membrane action potential. It was defined by Rosenfalck (1969).


(1) $$V^{m}(\zeta )=\left\{ \begin{array}{l} a\cdot \zeta^{3}\cdot e^{-\lambda \cdot \zeta }+b\;\zeta >0\\ \;b\;\zeta \leq 0, \end{array}\right.$$

where *a*∈ℝ^+^ and λ∈[0, 1] are shape defining parameters, *b*∈ℝ^−^ is the resting potential, and ζ is the myoelectric action potential local coordinate. In this study, the parameters of the myoelectric action potential are set to *a* = 96mV/mm (Farina and Merletti, 2001), *b* = −80mV (Ludin, 1969), and λ = 1/mm (Farina and Merletti, 2001).

Using the approximation of the membrane potential from Equation 1, a trans-membrane current density can be determined using the core conductor model as described by Taylor (1963). The core conductor model divides the electrically active cell membrane into segments. If the segment width is approaching zero, a continuous description of the trans-membrane current density is achieved as shown in Equation 2.


(2) $$\begin{array}{l} \begin{array}{c} I^{m}\left(\zeta \right)=\frac{a\cdot \zeta }{R^{e}+R^{i}}\cdot \left({\left(\lambda \cdot \zeta \right)}^{2}-6\lambda \cdot \zeta +6\right)\cdot e^{-\zeta \lambda }, \end{array} \end{array}$$

where *R*^*e*^ and *R*^*i*^ are the resistance per unit length within and outside the cell, respectively. The current source density will be used to calculate concentrated current sources, which in turn will be used as current sources for the electric field model of the muscle fiber.

A membrane region with negative ions flowing into the muscle fiber is considered to be a current source. If negative ions leave the muscle fiber within a membrane region, this region can be regarded as a current sink. This definition conforms with the technical definition of current sources and sinks. In the case of the trans-membrane current density described in Equation 2, there are three distinct regions (*S*_I_, *S*_II_, and *S*_III_), as also indicated in Figure 2B. Each region can be interpreted either as a distinct current source or sink (in Figure 2B, a source is represented by ⊕ and a sink by ⊖). If the current distribution within a section is concentrated in a single current (concentrated current source), it would be located at the center of the respective section. There are two positive regions (one at the beginning and the other at the end of the signal) and one negative region between them. In terms of current sources and sinks, this means a current source ⊕, followed by a sink ⊖ and another source ⊕.

In order to determine the concentrated sources, the bounds of each section (*S*_I_, *S*_II_, and *S*_III_) have to be known. The corresponding bounds are found by analyzing the roots of Equation 2. These roots are used to divide the current density into three sections with the following boundaries.


(3) $$S_{\text{I}}=\left[\begin{array}{c} 0\\ -\frac{\sqrt{3}-3}{\lambda } \end{array}\right],S_{\text{II}}=\left[\begin{array}{c} -\frac{\sqrt{3}-3}{\lambda }\\ \frac{\sqrt{3}+3}{\lambda } \end{array}\right],\text{and }S_{\text{III}}=\left[\begin{array}{l} \frac{\sqrt{3}+3}{\lambda }\\ \lim_{\upsilon \to \infty }\upsilon \end{array}\right].$$

The trans-membrane current per concentrated current source is now calculated using the definite integral of *I*^*m*^ in Equation 2 within each section using the boundaries of Equation 3. The general solution for the concentrated current source of a section *S*_*i*_ is


(4) $$\begin{array}{l} i_{i}^{m}=\int_{e_{i}}^{f_{i}}I^{m}(\zeta )\;d\zeta , \end{array}$$

with *f*_*i*_ and *e*_*i*_ being the section boundaries. The position of the concentrated current source on the ζ-axis is chosen to be at the ζ-coordinate of the centroid of a section.


(5) $$\begin{array}{l} c_{i}=\frac{{\left[\zeta \cdot \frac{\partial }{\partial \zeta }V^{m}(\zeta )\right]}_{e_{i}}^{f_{i}}-{\left[V^{m}(\zeta )\right]}_{e_{i}}^{f_{i}}}{{\left[\frac{\partial }{\partial \zeta }V^{m}(\zeta )\right]}_{e_{i}}^{f_{i}}}, \end{array}$$

where *c*_*i*_ is the position of the *i*th concentrated current source in the ζ-coordinate system.

The model of the concentrated current source, as introduced in Equations 4, 5, is stationary, thus neglecting the emergence at the motor end plate, propagation along the fiber, and disappearance at the tendon junctions at the ends of the muscle fiber. To incorporate these aspects, boundaries of muscle fiber and the traveling of the current source have to be taken into account.

When a muscle fiber is innervated, the myoelectric action potential is generated at the motor end plate, triggered by the neurotransmitter acetylcholine. As compared to the axial length of the muscle fiber, the motor end plate is small. Therefore, the borders of the motor end plate to the left and right of the voltage-gated membrane section are assumed to coincide (innervation point). The myoelectric action potential gradually emerges from this innervation point and excites the neighborhood on both sides. This results in two sets of concentrated current sources which then “travel” bidirectionally along the muscle fiber away from the innervation point.

When both sets of the concentrated current sources (the left and right sides of the innervation point) have fully emerged, there is no change in the shape of the current source curves until they reach the end of the muscle fiber. In this study, it is assumed that the myoelectric action potential (representing a set of concentrated current sources) travels with constant speed (*conduction velocity*) between the innervation point and the myotendinous junction (transition between muscle and tendon tissue). This assumption is based on an assumed homogeneous ion channel distribution along the muscle fiber.

Physiologically, the skeletal muscle fiber is attached to the tendon tissue at both ends. The tendon has no excitable cell membrane, thus the signal cannot propagate any further and disappears at the myotendinous junction. This disappearance is often observed as the *end effect* in the sEMG as a stationary signal component (Roberto Merletti, 2004, p. 181). In order to model the finiteness of the muscle fiber, in this study, the *sealed end* approach (Dumitru, 2000) is used.

The propagation of the concentrated current source for an infinite muscle fiber without an innervation point can be described by changing the axial position of the concentrated currents $i_{i}^{m}$ over time. In contrast, for a finite muscle fiber with an innervation point, the emergence at the innervation point and disappearance at the myotendinous junction have to be taken into account. Using the coordinate definition from Figure 2, the left tendon onset is set at *x* = 0, the innervation point at *x* = *P*^*IP*^, and the right tendon onset at *x* = *P*^*MF*^. The length of the muscle fiber is given by *P*^*MF*^, and *x* is the axial coordinate. There are two sets of concentrated current sources. One set travels in the direction of the left tendon and the other travels toward the right tendon.

The left signal emerges from the innervation point. Therefore, the source function has to be shifted to *x* = *P*^*IP*^, and the left side propagation is modeled with the translated coordinate, *x*^*l*^(ζ, *t*). This transformation is time dependent, as the current source travels along the muscle fiber.


(6) $$\begin{array}{l} x^{l}\left(\zeta ,t\right)=\zeta +P^{IP}-v\cdot t, \end{array}$$

where *v*≥0 is the conduction velocity of the muscle fiber. Equation 6 is rearranged to ζ and inserted into the function for the current density in Equation 6. Since *x*^*l*^(ζ, *t*) is the conversion from ζ to *x* coordinate, the superscript *l* can be omitted for the right hand side of Equation 7.


(7) $$\begin{array}{l} I^{m,l}\left(x,t\right)=I^{m}\left(x-\left[P^{IP}-v\cdot t\right]\right). \end{array}$$

The coordinate translation in Equation 6 should also be applied to the respective section limits.


(8) $$\begin{array}{ll} e_{i}^{l}\left(t\right)=x^{l}\left(e_{i},t\right) & =e_{i}+P^{IP}-v\cdot t\\ f_{i}^{l}\left(t\right)=x^{l}\left(f_{i},t\right) & =f_{i}+P^{IP}-v\cdot t. \end{array}$$

The finiteness of the muscle fiber is modeled for the emergence and disappearance by adjusting the section boundaries (*sealed end*, denoted by the superscript, fin).


(9) $$\begin{array}{l} e_{i}^{l,\text{fin}}(t)=\{\begin{array}{ll} P^{IP} & P^{IP}\leq e_{i}^{l}(t)\\ e_{i}^{l}(t) & 0<e_{i}^{l}(t)<P^{IP}\\ 0 & \text{otherwise} \end{array}\\ f_{i}^{l,\text{fin}}(t)=\left\{ \begin{array}{l} P^{IP}\;P^{IP}\leq f_{i}^{l}(t)\\ f_{i}^{l}(t)\;0<f_{i}^{l}(t)<P^{IP}.\\ \;0\text{ otherwise} \end{array}\right. \end{array}$$

As a result, the concentrated current source of a section *i*∈{I, II, III} and its position are as follows:


(10) $$\begin{array}{l} i_{i}^{m,l,\text{fin}}(t)=\int_{e_{i}^{l,\text{fin}}(t)}^{f_{i}^{l,\text{fin}}(t)}I^{m,l}(x,t)\;dx\\ c_{i}^{l,\text{fin}}(t)=\frac{1}{i_{i}^{m,l,\text{fin}}(t)}\cdot \int_{e_{i}^{l,\text{fin}}(t)}^{f_{i}^{l,\text{fin}}(t)}x\cdot I^{x,l}(x,t)\;dx. \end{array}$$

The signal that travels to the right is modeled similar to the left signal.


(11) $$\begin{array}{l} x^{r}\left(\zeta ,t\right)=\zeta +P^{IP}+v\cdot t. \end{array}$$

Like above, *x*^*r*^(ζ, *t*) is the conversion from ζ to *x* coordinate, and the superscript *r* can be omitted on the right hand side of Equation 12. This results in the description of the current density in Equation 12.


(12) $$\begin{array}{l} I^{m,r}\left(x,t\right)=I^{m}\left(-\left[x-\left(+P^{IP}+v\cdot t\right)\right]\right). \end{array}$$

The respective adaptation of the section boundaries is shown in Equation 13.


(13) $$\begin{array}{ll} e_{i}^{r}\left(t\right)=x^{r}\left(e_{i},t\right) & =e_{i}+P^{IP}+v\cdot t\\ f_{i}^{r}\left(t\right)=x^{r}\left(f_{i},t\right) & =f_{i}+P^{IP}+v\cdot t, \end{array}$$

and of the disappearing section boundaries in Equation 14.


(14) $$\begin{array}{l} e_{i}^{r,\text{fin}}(t)=\{\begin{array}{ll} P^{IP} & P^{IP}\geq e_{i}^{r}(t)\\ e_{i}^{r}(t) & P^{IP}<e_{i}^{r}(t)<P^{MF}\\ P^{MF} & \text{otherwise} \end{array}\\ f_{i}^{r,\text{fin}}(t)=\left\{ \begin{array}{l} P^{IP}\;P^{IP}\geq f_{i}^{r}(t)\\ f_{i}^{r}(t)\;P^{IP}<f_{i}^{r}(t)<P^{MF}.\\ P^{MF}\text{ otherwise} \end{array}\right. \end{array}$$

As a result, the concentrated current source of a section *i*∈{I, II, III} and its position are as follows:


(15) $$\begin{array}{l} i_{i}^{m,r,\text{fin}}(t)=\int_{e_{i}^{r,\text{fin}}(t)}^{f_{i}^{r,\text{fin}}(t)}I^{m,r}(x,t)\;dx\\ c_{i}^{r,\text{fin}}(t)=\frac{1}{i_{i}^{m,r,\text{fin}}(t)}\cdot \int_{e_{i}^{r,\text{fin}}(t)}^{f_{i}^{r,\text{fin}}(t)}x\cdot I^{m,r}(x,t)\;dx. \end{array}$$

#### 2.1.2. Muscle fiber electrode potential

To set up a simulation for the expected potentials that would be measurable at the skin surface with sEMG electrodes, the potential at an observation site outside the muscle fiber must be determined mathematically. As an extracellular field description, the model of muscle fiber in an unbounded, isotropic, uniform, and two dimensional volume conductor, according to Plonsey (1977), is used.


(16) $$\begin{array}{l} \phi (\vec{P})=\frac{1}{4\pi \sigma^{e}}\int_{-\infty }^{\infty }\frac{I^{m}(\zeta )}{r(\vec{P},\zeta )}d\zeta , \end{array}$$

where $\vec{P}$ is the point of observation and σ^*e*^ is the conductivity of extracellular medium. The distance to the signal source $r\left(\vec{P},\zeta \right)$ is measured to the center axis of the muscle fiber. The integral in Equation 16 is split into parts using the section boundaries as follows.


(17) $$\phi (\vec{P})=\frac{1}{4\pi \sigma^{e}}(\underset{=\;0}{\underset{︸}{\int_{-\infty }^{e_{\text{I}}^{-}}\frac{I^{m}(\zeta )}{r(\vec{P},\zeta )}\;d\zeta }}+\sum_{i\in \{\text{I},\text{II},\text{III}\}}\int_{e_{i}}^{f_{i}}\frac{I^{m}(\zeta )}{r(\vec{P},\zeta )}d\zeta ),$$

with $e_{\text{I}}^{-}=\underset{h\to 0}{\lim}\left(e_{\text{I}}-h\right)$. As *I*^*m*^(ζ) = 0 for all ζ <0, Equation 17 turns into


(18) $$\phi (\vec{P})=\frac{1}{4\pi \sigma^{e}}\sum_{i\in \{\text{I},\text{II},\text{III}\}}\int_{e_{i}}^{f_{i}}\frac{I^{ms}(\zeta )}{r(\vec{P},\zeta )}d\zeta .$$

**Figure 3:**
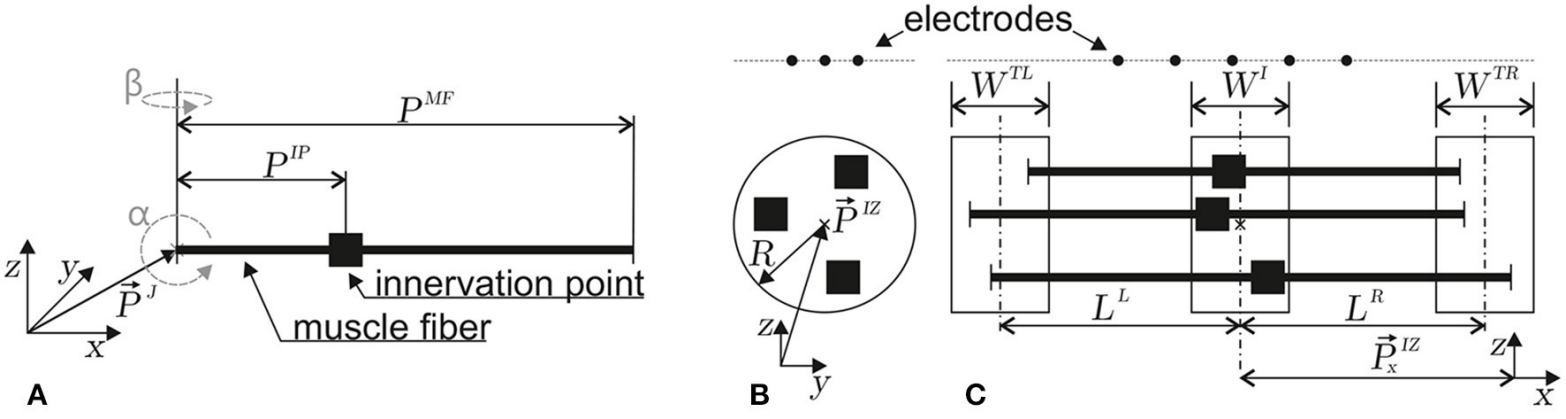
Depictions of the geometrical parameters of a muscle fiber **(A)** and a motor unit **(B, C)**. The innervation points are marked by ■. In **(A)**, the orientation (α, β) and position parameters (reference point at myotendinous junction ${\vec{P}}^{J}$, innervation point *P*^*IP*^, and fiber length *P*^*MF*^) of a single muscle fiber in three-dimensional space are defined. **(B)** Within a motor unit, the positions of innervation points are taken from a circular region within the *z, y* plane (center ${\vec{P}}^{IZ}$ and radius *R*). **(C)** Within the *z, x* plane, the left myotendinous junction point is taken from the *W*^*TL*^ region (the center of which has a distance *L*^*L*^ from the center of the *W*^*I*^ region). The right myotendinous junction point is taken from the *W*^*TR*^ region (the center of which has a distance *L*^*R*^ from the center of the *W*^*I*^ region). The x-coordinate (${\vec{P}}_{x}^{IZ}$) of the innervation point is taken from the *W*^*I*^ region. This type of motor unit parameterization is based on the work of Merletti et al. (1999).

In Equation 18, the observation point $\vec{P}$ is specified relative to the action potential local coordinate system ζ. However, in Equation 19, a coordinate transformation from the local coordinate system of an action potential to a global coordinate system, as defined in Figure 3A, is introduced. The observation point (i.e. electrode) in the global coordinate system is called ${\vec{P}}^{E}$. The concentrated current source per section in Equation 19 is assumed to be located at its distribution center, and finite muscle fiber conditions are assumed. In terms of an observation point in the global coordinate system, the potential field is modeled as follows for the concentrated current source traveling on the left side:


(19) $$\begin{array}{ll} \phi^{l}\left({\vec{P}}^{E},t_{k}\right) & =\frac{1}{4\pi \sigma^{e}}\underset{i\in \left\{\text{I},\text{II},\text{III}\right\}}{\sum }\frac{i_{i}^{m,l,\text{fin}}\left(t_{k}\right)}{r\left({\vec{P}}^{E},c_{i}^{l,\text{fin}},t_{k}\right)}, \end{array}$$

with


(20) $$\begin{array}{ll} r({\vec{P}}^{E},c_{i},t_{k}) & =\|c_{i}(t_{k})\left[\begin{array}{c} \cos\beta \cdot \cos\alpha \\ \sin\beta \cdot \cos\alpha \\ -\sin\alpha \end{array}\right]+{\vec{P}}^{MF}{-{\vec{P}}^{E}\|}_{2}\;. \end{array}$$

If the superscript l is replaced by r, equation 19 also applies to the right traveling concentrated current sources. Due to the assumption of a quasi static electric field, signal propagation is modeled in discrete steps (quasistatic, *t*_*k*+1_ = *t*_*k*_+Δ*t*). If the potential field generated by the right traveling current source is treated similarly and due to the linearity of the potential field, a complete field description for an unbounded volume conductor with a muscle fiber of finite length is given by


(21) $$\begin{array}{l} \phi \left({\vec{P}}^{E},t_{k}\right)=\phi^{l}\left({\vec{P}}^{E},t_{k}\right)+\phi^{r}\left({\vec{P}}^{E},t_{k}\right). \end{array}$$

After inserting the respective terms, this leads to


(22) $$\begin{array}{l} \begin{array}{cc} \phi \left({\vec{P}}^{E},t_{k}\right)\\ =\frac{1}{4\pi \sigma^{e}} & \left(\underset{i\in \left\{\text{I},\text{II},\text{III}\right\}}{\sum }\frac{i_{i}^{m,l,\text{fin}}\left(t_{k}\right)}{r\left({\vec{P}}^{E},c_{i}^{l,fin},t_{k}\right)}\;+\underset{i\in \left\{\text{I},\text{II},\text{III}\right\}}{\sum }\frac{i_{i}^{m,r,\text{fin}}\left(t_{k}\right)}{r\left({\vec{P}}^{E},c_{i}^{r,fin},t_{k}\right)}\right). \end{array} \end{array}$$

This description of a single muscle fiber electrode potential is used to model a motor unit electrode potential in the following section.

#### 2.1.3. Motor unit electrode potential and simulation setup

Each motor unit consists of multiple muscle fibers. The sum of electrode potentials of all muscle fibers Equation 22 at ${\vec{P}}^{E}$ gives the motor unit electrode potential at ${\vec{P}}^{E}$. The individual parameters of each muscle fiber depend on the motor unit configuration. The geometry of a motor unit is defined by the region where the innervation points of each muscle fiber are located, as well as the regions where the individual muscle fibers end. In this study, the muscle fibers are assumed to be parallel to each other (α = β = 0). The innervation points are drawn from a uniform distribution within the cylindrical volume defined by the parameters [*W*^*I*^, ${\vec{P}}^{IZ}$, and *R*], as shown in Figures 3B, C. The left end of a muscle fiber is drawn from a uniform distribution defined by [${\vec{P}}_{x}^{IZ}$, *L*^*L*^, and *W*^*TL*^]. Similarly, the right end is drawn from a uniform distribution defined by [${\vec{P}}_{x}^{IZ}$, *L*^*R*^, and *W*^*TR*^; Figure 3C]. The geometry constraints are drawn individually for each muscle fiber and are then transformed into muscle fiber parameters.

The amount of muscle fibers within a motor unit is assumed to non linearly depend on the relative motor neuron size as described by Enoka and Fuglevand (2001).


(23) $$\begin{array}{l} N^{\text{MF}}\left(i\right)=a\cdot \text{exp}\left(\frac{\text{ln}\left(R\right)}{N^{\text{MU}}}\cdot i\right)\;\text{with }i\in \left[0,N^{\text{MU}}\right], \end{array}$$

where *i*∈ℕ is the motor unit identifier. The smallest number of muscle fibers within a motor unit was assumed as *a* = 21, based on the findings from the *first dorsal interosseous muscle* as reported by Enoka and Fuglevand (2001). In accordance with the article on *biceps brachii* reported by Buchthal and Schmalbruch (1980), the total number of muscle fibers was chosen as *N*^MF, total^ = 580·10^3^ and the number of motor units was set to *N*^MU^ = 774. The parameter *R* was chosen such that the total amount of muscle fibers of all motor units is $\overset{N^{\text{MF},\text{total}}}{\underset{i=0}{\sum }}N^{\text{MF}}\left(i\right)=N^{\text{MF},\text{total}}$, which resulted in *R*≈188.6.

The motor unit conduction velocity seems to depend linearly on the force twitch rise time (Andreassen and Arendt-Nielsen, 1987). If in turn the twitch rise time is assumed to depend linearly on the motor unit size, the following description can be used to model the motor unit conduction velocity.


(24) $$\begin{array}{l} v^{\text{MU}}\left(i\right)=\frac{v_{\text{max}}-v_{\text{min}}}{N^{\text{MU}}-1}\cdot i+v_{\text{min}}. \end{array}$$

Here, $v_{\text{min}}=2.5\frac{\text{m}}{\text{s}}$ and $v_{\text{max}}=5.4\frac{\text{m}}{\text{s}}$ were assumed (Andreassen and Arendt-Nielsen, 1987). The conduction velocity of a muscle fiber within a motor unit *i* was drawn from the normal distribution given by


(25) $$\begin{array}{l} v^{\text{MF}}\left(i\right)\sim N\left(\mu =v^{\text{MU}}\left(i\right),\sigma =0.22\frac{m}{s}\right), \end{array}$$

where σ was taken from Farina et al. (2000).

In summary, the motor unit is parameterized by a set of geometry parameters (motor unit geometry set)


(26) $$\underset{\text{innervation zone}}{\underset{︸}{\{{\vec{P}}^{IZ},R,W^{I}}},\underset{\begin{array}{c} \text{left and right}\\ \text{myotendinous junction} \end{array}}{\underset{︸}{L^{L},L^{R},W^{TL},W^{TR}\}}}$$

and the amount of muscle fibers within a motor unit *N*^MU^, from which the conduction velocity of each muscle fiber is calculated.

### 2.2. Myoelectric simulation framwork - experimental setup

For the generation of a virtual electromyography data set, 48 motor units were simulated with varying parameters in six different motor unit geometry sets (see Equation 26 and Figure 3), and per set with eight different motor unit sizes (number of muscle fibers). The simulations were conducted with varying signal-to-noise ratios. The innervation zone parameters (the left side of Equation 26) were constant for all simulated motor units and were chosen arbitrarily in plausible ranges.


(27) $$\begin{array}{l} W^{I}=2\text{ cm }R\;=\sqrt{\frac{10\;{\text{cm}}^{2}}{\pi }}\;{\vec{P}}^{IZ}={\left[0,0,0\right]}^{T}. \end{array}$$

The widths of the myotendinous junction region (the right side of Equation 26) were fixed to *W*^*TL*^ = *W*^*TR*^ = 0.5cm for all simulated motor units. However, the shape parameters affecting the position of the myotendinous junction regions were varied. The distance between the left and right myotendinous junction regions *L* = *L*^*L*^+*L*^*R*^ was either set to *L* = 15cm or *L* = 19cm. For both *L* configurations, three sets of *L*^*L*^ and *L*^*R*^ were simulated.


(28) $$\begin{array}{l} \;L^{L}=L\cdot g^{L}\\ \;L^{R}=L\cdot g^{R},\\ \text{where}({\text{g}}^{\text{L}},{\text{g}}^{\text{R}})\in \{(\text{0}.\text{5},\text{0}.\text{5}),(\text{0}.\text{7},\text{0}.\text{3}),(\text{0}.\text{25},\text{0}.\text{75})\}. \end{array}$$

For each of the six motor unit geometry sets, eight motor units with different sizes were generated. The amount of muscle fibers within each of the eight motor units was determined by Equation 23. The motor unit id *i* also refers to the motor unit size.


(29) $$\begin{array}{l} i\in 400,450,500,\ldots ,750. \end{array}$$

The conduction velocity of each individual muscle fiber was set as described in Equations 24, 25 based on the amount of muscle fibers within a motor unit.

For the electrode configuration, a linear electrode array with a 5mm inter electrode distance (IED) was chosen. The electrode array was placed 2cm above the innervation zone center ${\vec{P}}^{IZ}$. The array consisted of *N*_elec_ = 68 electrodes. In total, 20 electrode arrays per motor unit, each with 68 electrodes, were simulated. The electrode arrays differed in an x-offset.


(30) $$\begin{array}{l} {\vec{P}}_{e}^{E}=\left[\begin{array}{c} -17\text{ cm}+(e\;\cdot \;0.5\text{ cm}+\text{offset})\\ 0\text{ cm}\\ 2\text{ cm} \end{array}\right]\;,\text{ where }e\in [0,N_{\text{elec}}-1],\\ \text{ where offset}\in \left\{n\cdot \frac{0.5\text{ cm}}{20}n\in [0,19]\right\} \end{array}$$

The index *e* is the index of the mono polar electrode potential within each array. The algorithm presented in this study assumes a double differential electrode configuration. The double differential potential is calculated based on three neighboring mono polar electrode potentials as follows:


(31) $$\begin{array}{l} \begin{array}{c} \phi_{DD}({\vec{P}}_{DD},t_{k})=(\phi ({\vec{P}}_{DD};t_{k})-\phi ({\vec{P}}_{DD+1};t_{k}))\\ \;-(\phi ({\vec{P}}_{DD+1};t_{k})-\phi ({\vec{P}}_{DD+2};t_{k})),\\ \text{ where }DD\in [0,65]. \end{array} \end{array}$$

The calculated double differential potentials were then located at the center electrode of each triplet.


(32) $$\begin{array}{l} {\vec{P}}_{DD}={\vec{P}}_{e=DD+1,}^{E}\;\text{where }DD\in \left[0,65\right]. \end{array}$$

**Figure 4:**
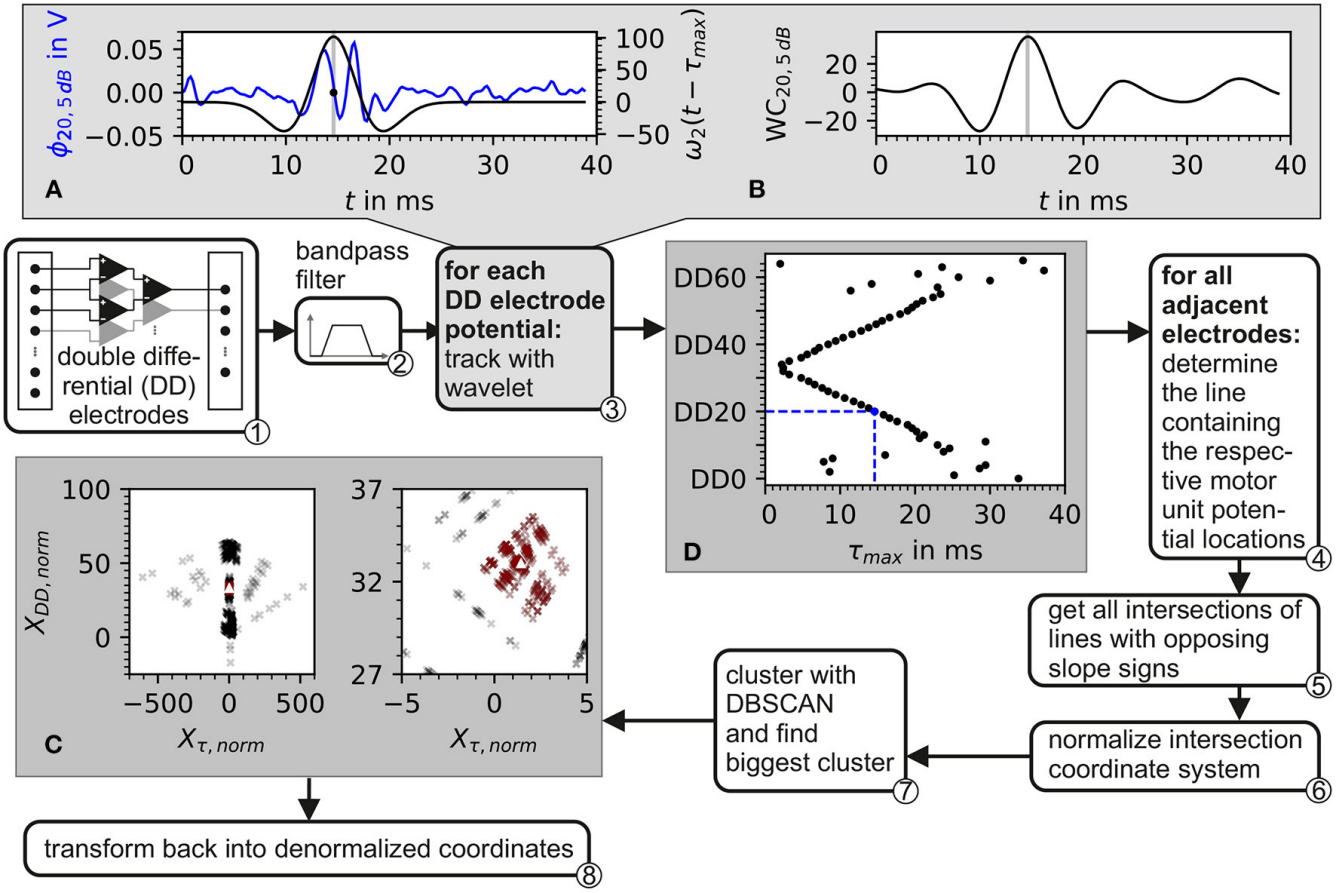
Block diagram of the algorithm for the estimation of the center of the innervation zone. Depiction also contains plots of intermediate data from inside the algorithm. ① The input consists of recordings from a double differential electrode configuration. ② In preprocessing, the data is bandpass filtered. ③ Each double differential electrode potential is correlated with the wavelet described in Equation 37. The plot in **(A)** shows the electrode potential of electrode DD20 represented in blue and the wavelet with the optimal phase represented in black. In **(B)**, the correlation of the wavelet with electrode DD20 is shown. The gray line in **(A, B)** marks the optimal wavelet position in time. In **(D)**, the optimal wavelet time delay τ for each double differential electrode is shown as black dots. ④ For each possible pair of these optimal wavelet time delays, a line is calculated. ⑤ From these lines, all intersections are determined. After the normalization of the intersection points w.r.t. inter electrode distance ⑥, the intersections are clustered with the DBSCAN algorithm ⑦. The biggest cluster is displayed in **(C)** as red crosses and then used to estimate the center of the innervation zone which is marked by the white triangle. ⑧ Transforms back to denormalized coordinates.

The electrode triplets were chosen as shown in ① of Figure 4.

To simulate varying noise levels, Gaussian noise was added to the double differential motor unit electrode potentials. The variance of the random Gaussian noise process is estimated based on a given signal-to-noise ratio.


(33) $$\begin{array}{l} \text{SNR}=10\log_{10}\left(\frac{P_{\text{signal}}}{P_{\text{noise}}}\right). \end{array}$$

The double differential motor unit electrode potential, as defined in Equation 31, is a time discrete signal. Therefore, the added noise signal will also be a time discrete signal. As a result, Equation 33 turns into


(34) $$\begin{array}{l} \text{SNR}=10\log_{10}\left((\underset{\text{signal power }P_{\text{signal}}}{\underset{︸}{\frac{1}{N_{t}}\sum_{k=1}^{N_{t}}{\left|\phi \left({\vec{P}}_{e}^{E},t_{k}\right)\right|}^{2}}})\cdot {\left(\underset{\approx \sigma^{2}}{\underset{︸}{\frac{1}{N_{t}}\sum_{k=1}^{N_{t}}{\left|e(t_{k})\right|}^{2}}}\right)}^{-1}\right),\\ \text{ with }e(t_{k})\sim N\left(\mu =0,\sigma \right), \end{array}$$

where σ is the uncorrected standard deviation of the additive noise and *N*_*t*_ is the number of simulated time steps. Equation 34 is then rearranged to σ.


(35) $$\begin{array}{l} \sigma \left(\text{SNR},P_{\text{signal}}\right)=\sqrt{\frac{P_{\text{signal}}}{10^{\text{SNR}\cdot 0.1}}}. \end{array}$$

All double differential potentials are stored in an array of size $N^{DD}\times N_{t}$ during simulation. For each double differential signal over time, the signal power *P*_signal_ (cmp. Equation 34) is calculated. The median of all signal powers ${\tilde{P}}_{\text{signal}}$ in the array is then determined. Based on this, the standard deviation $\sigma \left(\text{SNR},{\tilde{P}}_{\text{signal}}\right)$ of the noise is obtained according to Equation 35. For each SNR, Gaussian noise values are then drawn from that distribution and added to all potentials in the array.

The resulting double differential potentials with added Gaussian noise are then defined as


(36) $$\begin{array}{l} \begin{array}{c} \phi_{DD,\text{SNR}}\left({\vec{P}}_{DD}^{E},t_{k}\right)=\phi_{DD}\left({\vec{P}}_{DD}^{E},t_{k}\right)+e\left(t_{k}\right),\\ \text{with }e\left(t_{k}\right)\sim N\left(\mu =0,\sigma \left(\text{SNR},{\tilde{P}}_{\text{signal}}\right)\right). \end{array} \end{array}$$

In this simulation, the double differential potentials over time are generated with a sampling frequency of *f*_*s*_ = 5kHz. The simulation duration is set such that the action potential of the longest muscle fiber reaches the end of the respective fiber. This results in a simulation time of *T*_sim_ = 38.8ms.

### 2.3. Algorithm for the estimation of the center of the innervation zone center

The estimation of the center of the innervation zone is based on tracking the motor unit potential in a double differential electrode array configuration. The basic idea of the algorithm is that the motor unit potential is detected on both sides of the center of the innervation zone with at least two electrodes per side. From that, the position of the center of the innervation zone on the electrode array can be estimated. Ideally, two electrodes per side of the motor unit's center of the innervation zone would be sufficient. However, the detection of the motor unit potential is subject to inaccuracies due to shape changes of the motor unit potential caused by noise and varying conduction velocities. Here, an electrode array is used which is placed in the general orientation of the skeletal muscle fibers covering a sufficient area of the muscle to likely include the center of the innervation zone.

The proposed algorithm for the estimation of the center of the innervation zone assumes a linear electrode array with double differential electrode potentials. The algorithm consists of seven computational steps. These steps are displayed in Figure 4 as a block diagram, where ① represents the double differential electrode array input. The computational steps are marked with ② to ⑧ in the figure. The sub figures of Figures 4A–C display examples of intermediate results of critical algorithm step outputs.

② The input data was filtered with a Butterworth bandpass filter. The filter was implemented with 2 second-order sections. The cutoff frequencies were *f*_1_ = 4Hz and *f*_2_ = 500Hz. The cutoff frequencies were designed to safely maintain the EMG frequencies encountered in real sEMG measurements. The high pass cutoff frequency was chosen to also include low motor unit firing frequencies starting at ≈5Hz (Conwit et al., 1998). The shape defining frequencies of a motor unit potential are ensured to be present in the filtered signal by following the SENIAM low pass filter recommendations for sEMGs as reported by Roberto Merletti (2004).

③ The position of the motor unit potential is estimated by the convolution of a wavelet with the electrode potential over time. The motor unit potential is located at that point in time, at which the convolution is maximum. As wavelet, the Hermite-Rodriguez series expansion was selected, which is a Hermite Polynomial scaled and weighted with a Gaussian function (Conte et al., 1994). The third Hermite Polynomial is used as proposed by Farina et al. (2000). This results in the following wavelet description.


(37) $$\begin{array}{l} \omega_{2}\left(t,\lambda \right)=\frac{1}{\sqrt{2^{2}\cdot 2!}}\cdot H_{2}\left(\frac{t}{\lambda }\right)\cdot \frac{1}{\sqrt{\pi }\cdot \lambda }\cdot \text{exp}\left(-\frac{t^{2}}{\lambda^{2}}\right),\\ \text{ with }H_{\text{2}}(x)\text{ = }4x^{2}\;-\;2, \end{array}$$

and with λ being the width parameter of the wavelet. The wavelet ω_2_ is convoluted with each of the discrete double differential motor unit potentials at all time points *t*_*k*_. The maximum of the convolution denotes the point in time where the wavelet matches optimally to the course of the electrode potential as shown in Figures 4A, B. This optimal time point was determined for each motor unit configuration, each double differential electrode *DD*, and signal-to-noise ratio SNR as follows:


(38) $$\begin{array}{l} \tau_{\text{max},DD,\text{SNR}}=\underset{n\in \left[1,N_{t}\right]}{\text{arg max}}\underset{{\text{wavelet convolution WC}}_{DD,\text{SNR}}}{\underset{︸}{\left(\phi_{DD,\text{SNR}}\left[k\right]*\omega_{2}\left[k\right]\right)\left(t_{n}\right)}}. \end{array}$$

An example of the wavelet matching the electrode potential is shown in Figure 4A. The optimal wavelet position is marked by a gray line. In Figure 4B, the wavelet signal convolution WC over the whole simulation time is depicted. The result is an assumed motor unit potential position in time for each double differential electrode triplet τ_max, *DD*, SNR_. This position is further expressed as a vector. The subscript SNR is omitted in order to simplify the following equations. It has to be considered that all operations were still carried out for each simulated signal-to-noise ratio.


(39) $$\begin{array}{l} {\vec{T}}_{DD}={\left[\begin{array}{cc} \tau_{\text{max},DD} & DD \end{array}\right]}^{T}. \end{array}$$

④ Position pairs of motor unit potentials are formed for each position vector $\vec{T}$ with its subsequent neighbor based on the electrode id *DD*.


(40) $$\begin{array}{l} S_{P}=\left\{{\vec{T}}_{P},{\vec{T}}_{P+1}\right\},\;\text{where}P\in \left[0,DD-1\right]. \end{array}$$

For each of these pairs *S*_*P*_, parameters of a line crossing both points of the pair were calculated. Each line is described as


(41) $$\begin{array}{l} DD=m_{P}\cdot \tau_{\text{max},DD}+b_{P},\;\text{where}DD\in \left\{P,P+1\right\},\; \end{array}$$

with *m*_*P*_ and *b*_*P*_ as line parameters. If these line parameters do not exist as a real value, then the position pair is undefined.

⑤ For all existing position pairs *S*_*P*_, all possible intersections of lines with opposite slope signs are calculated. These intersection points are given by


(42) $$\begin{array}{l} {\vec{X}}_{m}={\left[\begin{array}{cc} X_{\tau_{m}} & X_{{DD}_{m}} \end{array}\right]}^{T}, \end{array}$$

with *m* being the intersection index.

⑥ The intersection points ${\vec{X}}_{m}$ are normalized.


(43) $$\begin{array}{l} {\vec{X}}_{m,\text{norm}}=\underset{N}{\underset{︸}{\left[\begin{array}{cc} 1 & 0\\ 0 & \frac{v_{\text{e}}}{\text{IED}} \end{array}\right]}}{\vec{X}}_{m}, \end{array}$$

where *v*_e_ is the expected conduction velocity (in this study, it is set to $4\frac{\text{m}}{\text{s}}$), and IED is the inter electrode distance (in this study, it is set to 5mm, see Equation 30). An example of normalized intersection points is shown as black crosses in Figure 4C. The individual intersection points are plotted with a transparency of 20%.

⑦ The normalized intersection points are clustered with the density-based clustering algorithm DBSCAN. The scikit-learn (v1.2.0) implementation of DBSCAN was used (Pedregosa et al., 2011). The clustering algorithm DBSCAN is configured with a minimal number of data points within a cluster of three. The density parameter ϵ remains a free parameter of the presented algorithm for the estimation of the center of the innervation zone. The biggest cluster is selected (red data points in Figure 4C); of which, the mean intersection point is calculated, which is named ${\vec{IC}}_{\text{norm}}$ (white triangle in Figure 4C).

⑧ Finally, ${\vec{IC}}_{\text{norm}}$ is denormalized.


(44) $$\begin{array}{l} \vec{IC}=N^{-1}{\vec{IC}}_{\text{norm}}. \end{array}$$

The estimation of the center of the innervation zone presented in this study has two parameters that remain free to be chosen by the user. These are the wavelet width parameter λ and the DBSCAN density parameter ϵ. These parameters are explored by parameter variation. To compare parameter settings, a metric is required. In terms of the performance of the algorithm, there are two general ways in which the algorithm can perform better or worse. The first is a statistical measure of prediction accuracy, i.e., the absolute mean error (MAE) between prediction and the known mean innervation point of a motor unit. The second is to determine whether the algorithm was able to predict the center of an innervation zone or not. To facilitate a comparison with the MAE, the amount of motor unit innervation zones that could not be estimated *N*_ne_ was used. If the center of the innervation zone could not be estimated at least once, the MAE is undefined.

Depending on the use case, it might be desirable to optimize the algorithm for high prediction accuracy, even if this comes at the expense of a low number of motor unit potentials that can be tracked. Therefore, an error score combining the MAE and *N*_ne_ is defined.


(45) $$\begin{array}{l} \text{ES}\left(\alpha \right)\;=0.5\cdot \left(\alpha \;\cdot \;\frac{\text{MAE}}{\text{IED }\cdot \;0.5}+(1-\alpha )\cdot \frac{N_{\text{ne}}}{N_{\text{total}}}\right),\;\\ \text{ where }0\leq \alpha \leq 1. \end{array}$$

The error score weight α can be used to set a trade-off between accuracy (small MAE) and robustness (small *N*_*ne*_). A high value for α corresponds to a strong emphasis on accuracy and a low emphasis on robustness and vice versa for a low value of α. In order to analyze the error score ES in the parameter space (λ, ϵ) of the algorithm, a parameter variation was conducted with a constant α = 0.5, and at four different signal-to-noise levels SNR ∈{−5, 0, 5, and 10 dB}. This parameter variation was then repeated by prioritizing the algorithm's robustness and accuracy, by choosing α = 0.25 and α = 0.75, respectively.

Additionally, the absolute error (AE) distribution and the amount of center positions of the innervation zone that were not tracked (N_*ne*_) were examined for six parameter sets over input signals with SNR's ranging from −10 to 20 dB in 5 dB increments. The parameter sets were chosen as the optimal parameters in the case of input signal SNR = 5 dB with an α∈{0.25, 0.5, 0.75} and in the case of an input signal SNR∈{0, 5, and 10 dB} with an α = 0.5.

## 3. Results

The presented algorithm for finding the center of the innervation zone was tested using simulated electromyography data with varying degrees of noise. First, the simulation results are presented and then the accuracy and robustness of the algorithm are examined. Configuration files for the EMG simulator (Mechtenberg, 2023) and the parameter optimization results are available on zenodo (https://doi.org/10.5281/zenodo.8056478).

### 3.1. Motor unit potential simulation

As described in Section 2.2, the simulation was set up with six motor unit geometry sets. For each motor unit geometry set, eight motor units of different motor unit sizes (e.g., with different numbers of muscle fibers) were randomly generated and used for simulation. With an average of 1, 368 muscle fibers per motor unit, a total of 65, 664 muscle fibers were simulated (6·8·1, 368). For each electrode in an array of 68 electrodes, 195 time steps of 0.2ms were calculated, resulting in a simulation of 39ms per electrode. The computational time for the simulation of a single muscle fiber for all 68 electrodes was 7.8ms on an Intel(R) Xeon(R) W-2295 processor. Most of the computation time was consumed for exporting the simulation results of each muscle fiber to the hard drive.

**Figure 5:**
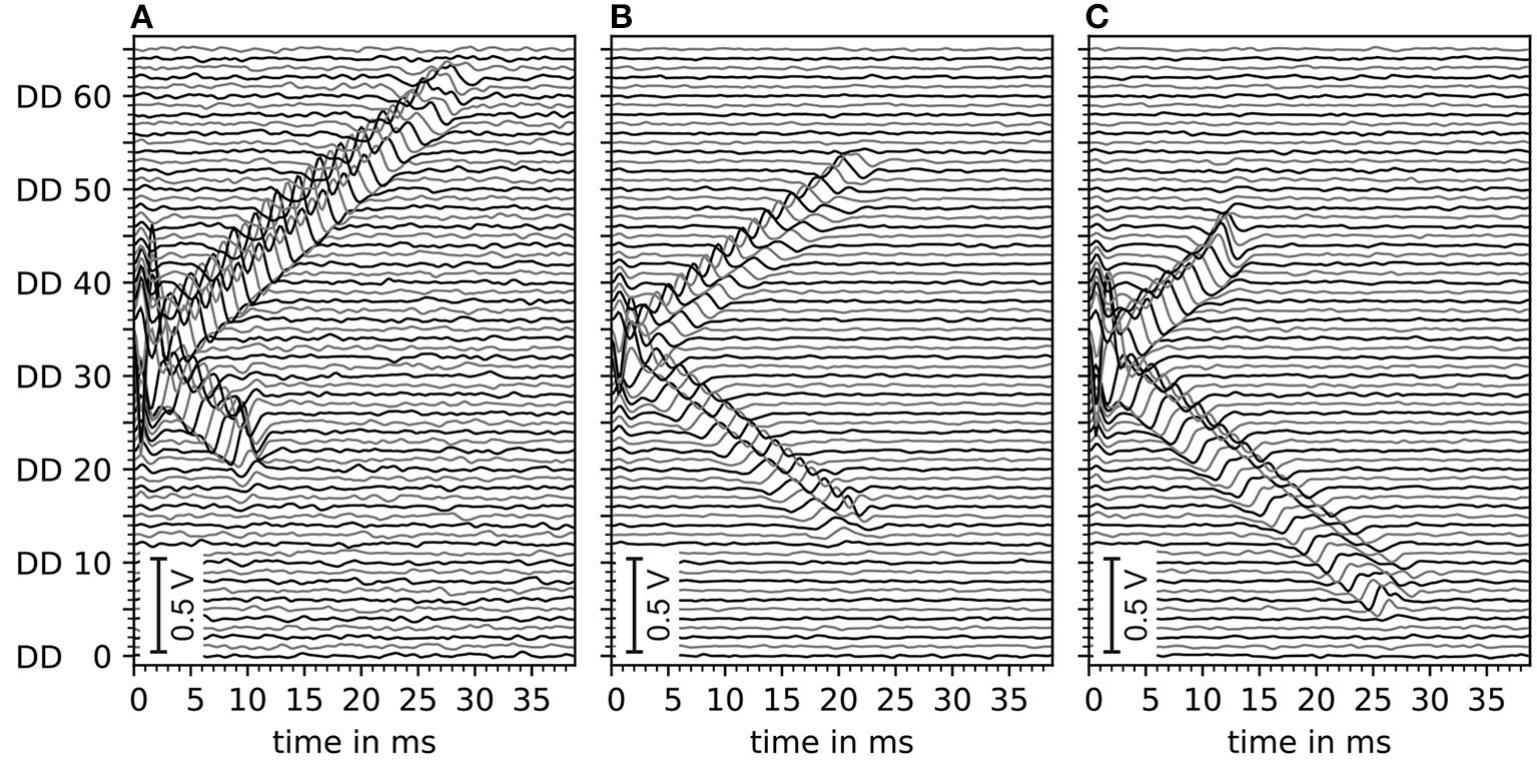
Depiction of exemplary motor unit electrode potentials of three different motor unit configurations, all with innervation zone center at electrode DD 33 (with DD being the double differential electrode id). **(A)** Distance to the left myotendinous junction *L*^*L*^ short and distance to the right myotendinous junction *L*^*R*^ long (ratio 25:75). **(B)** Both distances to myotendinous junctions are equal. **(C)** Same as **(A)** but mirrored (ratio changed to 70:30). All signals contain a Gaussian noise of an estimated 5 dB signal-to-noise ratio. Data is filtered by a digital Butterworth bandpass filter consisting of 2 second-order sections, where the cutoff frequencies are *f*_1_ = 4Hz and *f*_2_ = 500Hz.

The motor unit potential is the sum of electrode potentials of all muscle fibers. An example of motor unit simulation results are shown in Figure 5. The sub figures (A–C) show double differential electrode potentials for three different motor unit configurations with different distances (*L*^*L*^, *L*^*R*^) from the center of the innervation zone to the myotendinous junctions. Gaussian noise of 5 dB was added to the double differential signals before these, where bandpass was filtered as described in Section 2.3.

**Figure 6:**
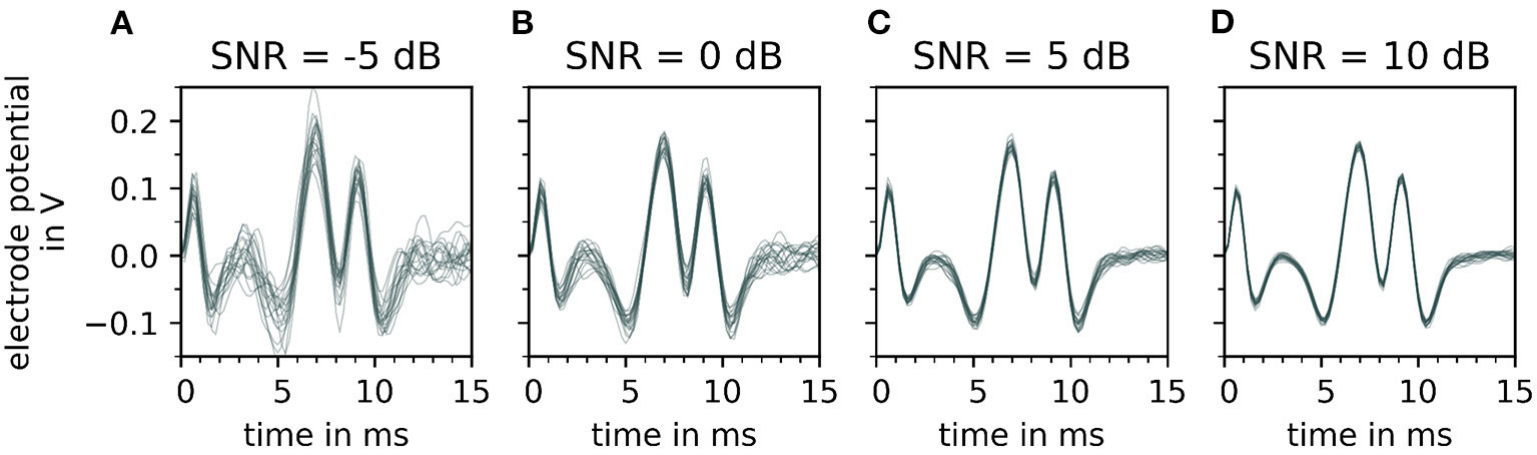
Depiction of a single motor unit electrode potential for varying signal-to-noise ratios (SNR), after bandpass filtering. From **(A–D)**, the signal-to-noise ratio increases, which means that the fraction of noise gets smaller. For each signal to noise ratio, 20 error signals are generated and added to the simulated signal. Each noisy electrode potential of motor unit is plotted over time on the top of each other with a constant transparency (30%). Note that with decreasing signal-to-noise ratio [from **(D–A)**] the extreme points of the electrode potentials of the motor unit electrode potentials vary strongly which influences the fit of the wavelet.

The effect of varying signal-to-noise levels on the filtered electrode potentials is demonstrated in Figure 6 with an increasing SNR∈{−5, 0, 5, and 10 dB} from (A–D). Whereas, the change in the peak shape of motor unit potential is noted with decreasing signal-to-noise level (D–A).

### 3.2. The estimation of the center of the innervation zone - robustness vs. quality for different SNRs

The goal of the development of the algorithm for estimating the center of the innervation zone was to achieve an algorithm that can be adapted to different noise conditions using a small set of parameters depending on the quality demands of the use case. The algorithm was designed to either achieve high accuracy in determining the center position of the innervation zone or to achieve high robustness to noise, at the expense of accuracy.

**Figure 7:**
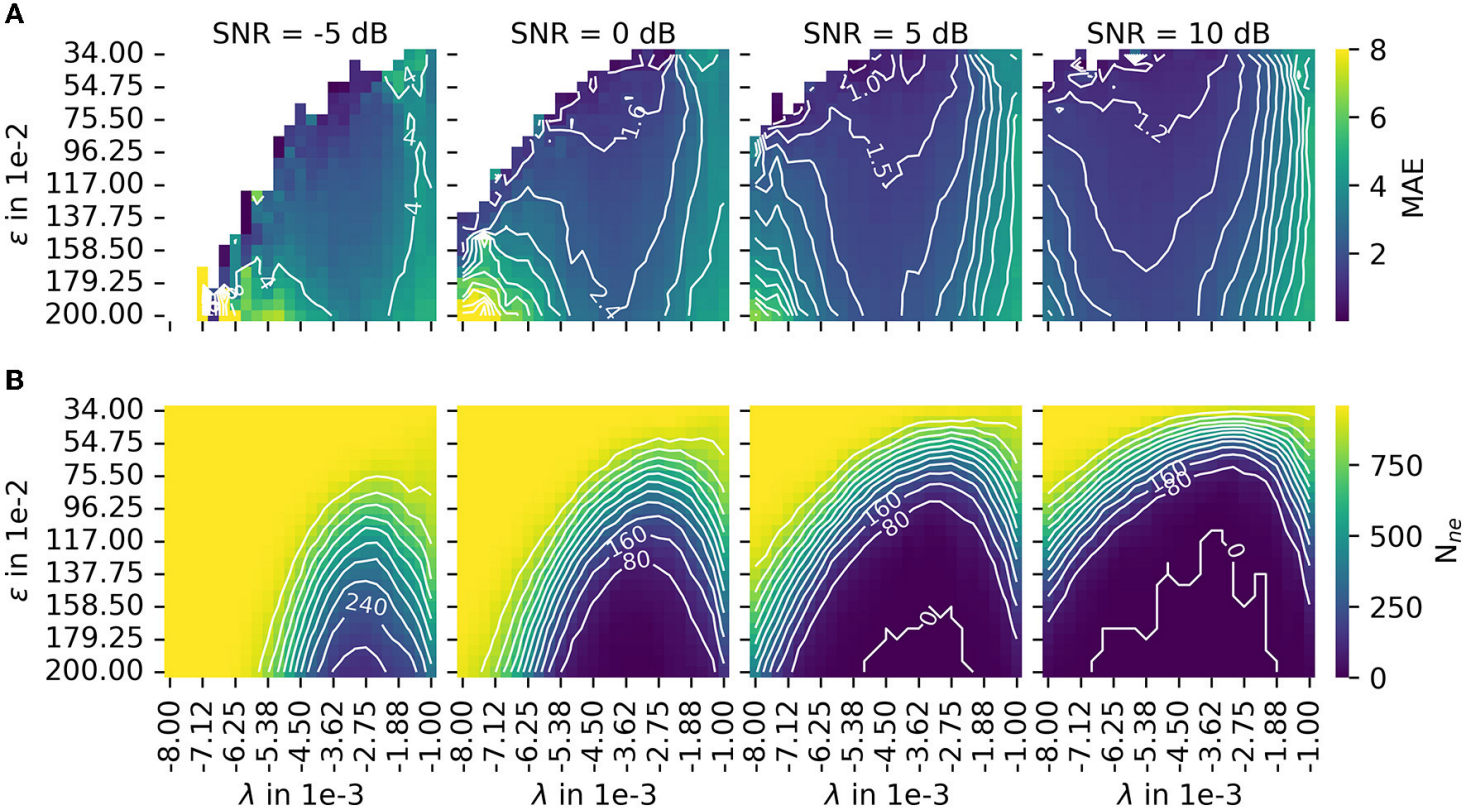
The results of the parameter variation are shown for the mean absolute error (MAE) in **(A)** and for the number of untrackable motor unit innervation zone centers *N*_*ne*_ in **(B)**. The leftmost column shows results for the lowest SNR = −5 dB. With each additional column, the SNR increases by 5 dB. Both measures, MAE and *N*_*ne*_, are normalized before they are combined to the error score *ES*(α), as defined in Equation 45. The combined error score in the case of varying weights α is shown in this figure.

**Figure 8:**
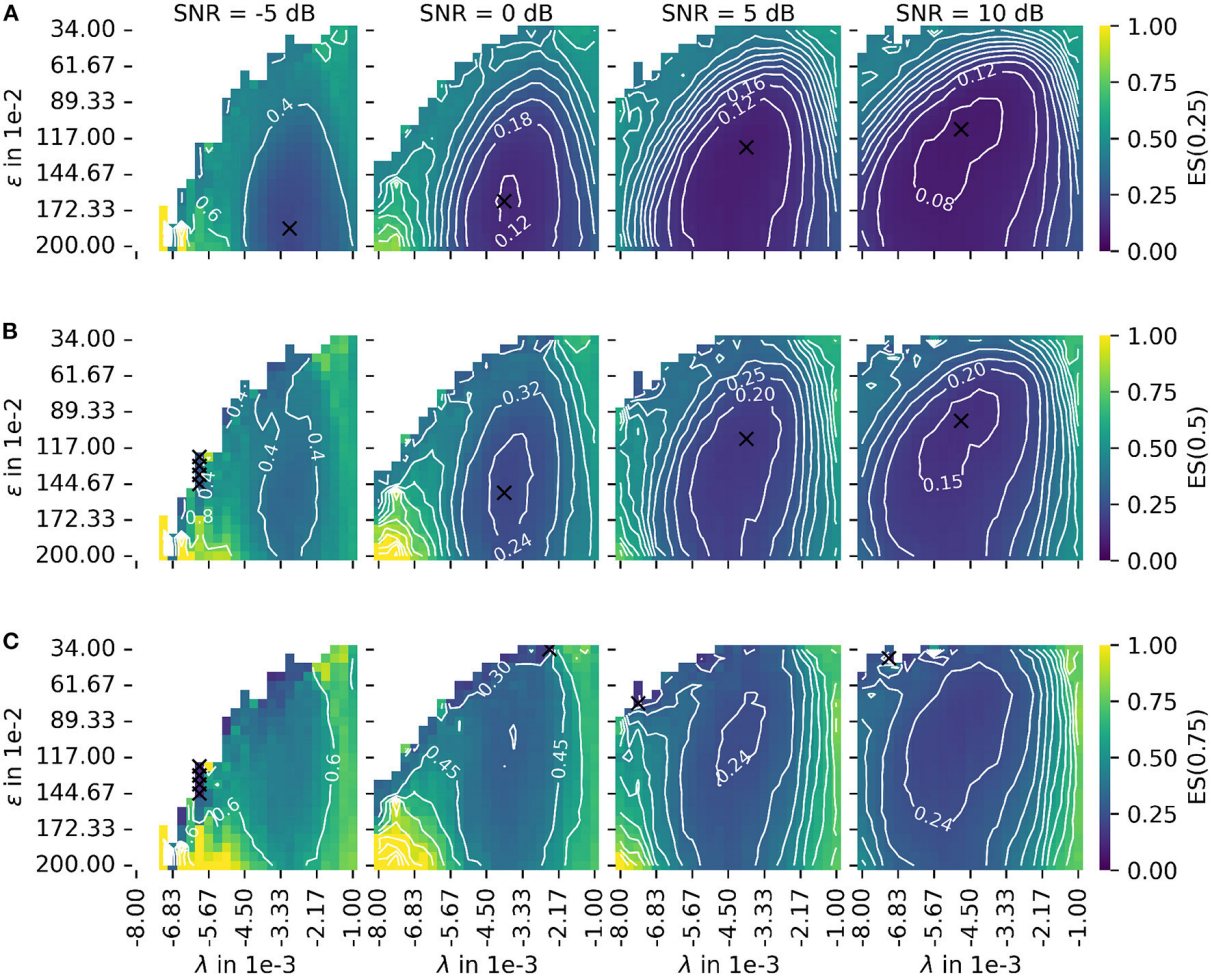
Depiction of the error score ES(α) as proposed in Equation 45 for different signal-to-noise ratios (SNR). The leftmost column shows results for the lowest SNR = −5 dB. With each additional column, the SNR increases by 5 dB. **(A)** The plot of the error score for α = 0.25 (stronger emphasis on robustness). **(B)** The plot of the error score for α = 0.5 (equal emphasis on robustness and accuracy). **(C)** The plot of the error score for α = 0.75 (stronger emphasis on accuracy). The parameter λ configures the wavelet width and the parameter ϵ defines the cluster density of the algorithm for the estimation of the center of the innervation zone. The color encodes the error score ES(α). A darker blue corresponds to a lower error score, and a lighter yellow corresponds to a higher error score. For white spots, the error score is undefined. For all plots, the optimal parameter set is marked with a black cross.

In Section 2.3, MAE was introduced as a measure for accuracy. The number of motor unit innervation centers that could not be identified (*N*_*ne*_) was introduced as the measure for the robustness of the algorithm (Details of MAE and *N*_*ne*_ are shown in Figure 7). To configure a trade off between accuracy and robustness, a new error score ES(α) was proposed in Equation 45. The parameter α is used to weigh the influence of the individual error measures, MAE and *N*_*ne*_. A parameter variation was conducted with a constant α = 0.5 and four different signal-to-noise levels (SNR) as depicted in Figure 8B. The parameters λ (wavelet width parameter) and ϵ (clustering density parameter) are plotted on the abscissa and ordinate, respectively. In the leftmost column, the results are shown for the lowest SNR = −5 dB. With each additional column, the SNR increases by 5 dB.

The color encodes the error score ES(α). A darker blue corresponds to a lower error score, and a lighter yellow corresponds to a higher error score. For white spots, the error score is undefined. For each SNR, the optimal parameter is marked with a black cross. Figure 8A shows the same as (B) but for a constant α = 0.25 (stronger emphasis on robustness). Figure 8C shows the same as (B) but for a constant α = 0.75 (stronger emphasis on accuracy). As a result, there are two factors determining the optimal mode of operation. These are the error score weight α and the input signal SNR for which the algorithm is used.

**Figure 9:**
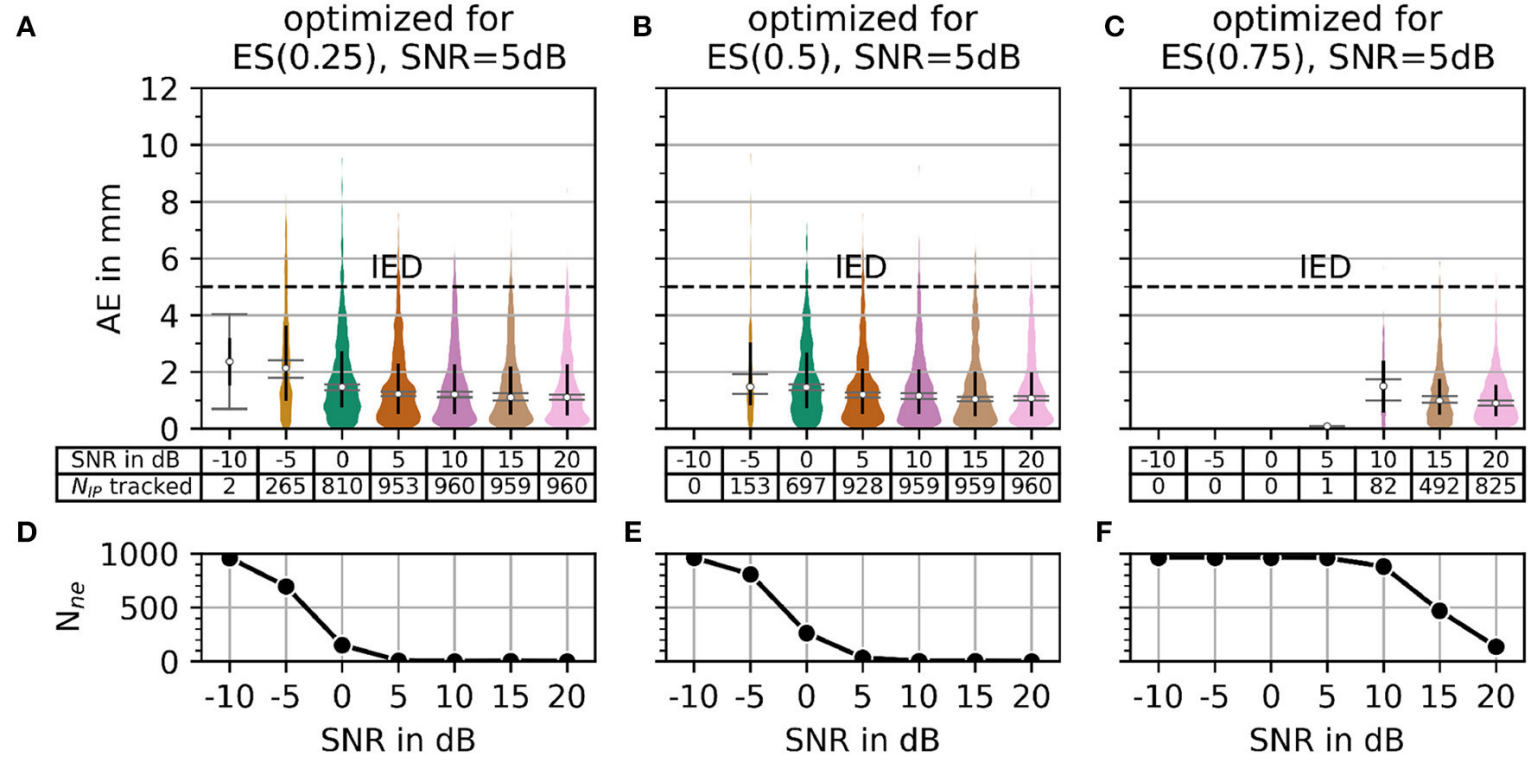
The plot of absolute error AE (in mm) and the number of untracked innervation centers for a selected parameterization from the SNR = 5 dB column of Figure 7. Column **(A, B)** shows the results for the optimal parameter set (black cross in Figure 7) for α = 0.25. Columns **(C, D)** and **(E, F)** show the same as the column **(A, B)** but for α = 0.5 and α = 0.75, respectively.

Regarding the question of which absolute numbers (accuracy in mm and the number of actually tracked innervation centers) belong to the error scores from Figure 8, the representation in Figure 9 was created. The third column in Figure 8 (error scores for SNR = 5 dB) are used to determine the optimal parameter sets for the results in Figure 9. Figure 9 shows the absolute error AE (in mm) and the number of untracked centers of the innervation zone *N*_*ne*_ belonging to an optimal parameter set (black cross in Figure 8). The MAE is plotted as a white dot per SNR category (gray whiskers represent the standard deviation of the MAE, and 0.25 and 0.75 percentiles are drawn as black lines). Column (A, D) shows this for ES(0, 25), (B, E) for ES(0.5), and (C, F) for ES(0.75). It can be seen that all MAE are below the inter electrode distance (IED). With a strong weightage on the accuracy [column (C, F)], significantly fewer motor units are tracked [no outliers above the IED line as opposed to columns (A, D) and (B, E)].

**Figure 10:**
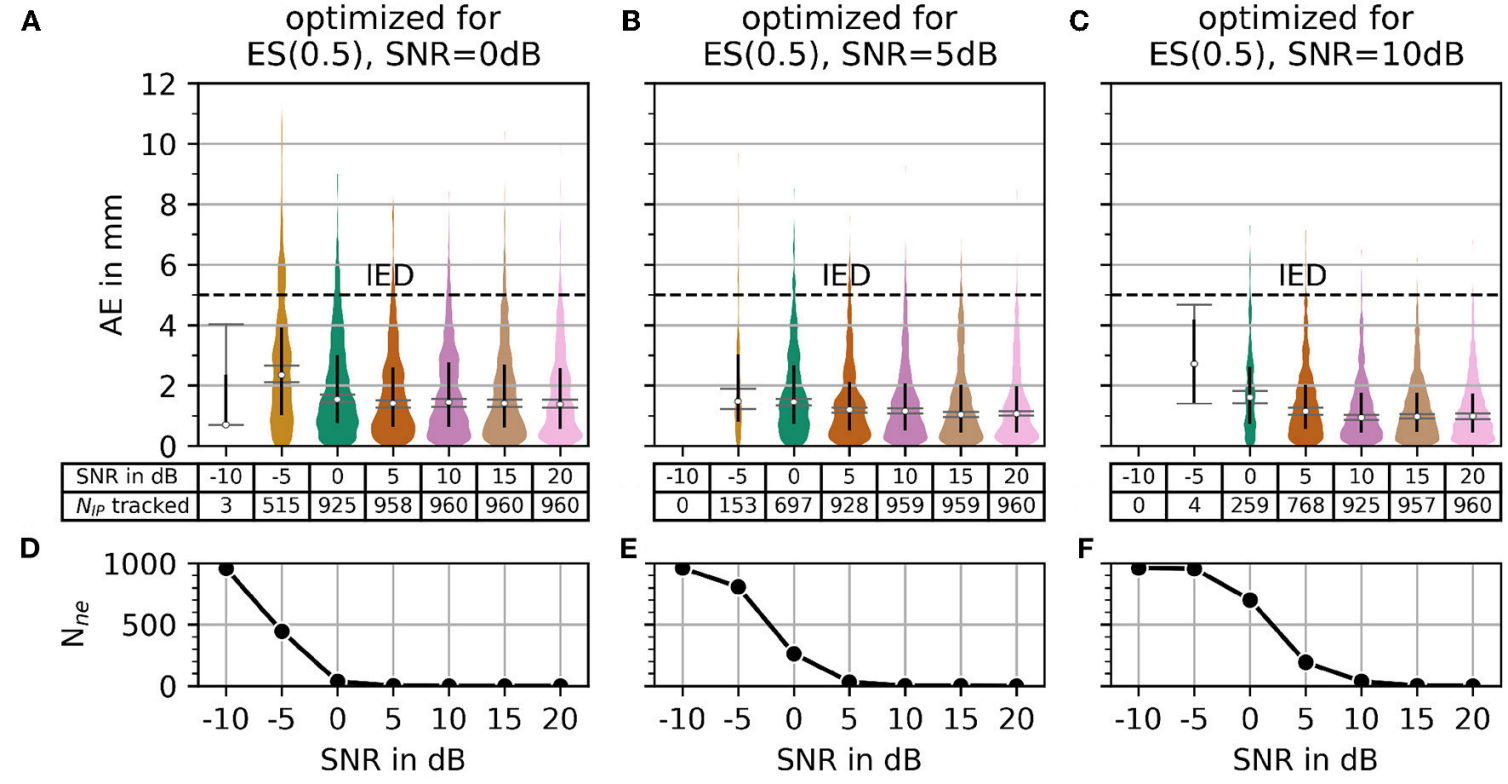
The plot of absolute error AE (in mm) and the number of untracked innervation centers for a selected parameterization from the α = 0.5 row of Figure 7. Column **(A, B)** shows the results for the optimal parameter set (black cross in Figure 7) for SNR = 0 dB. Columns **(C, D)** and **(E, F)** show the same as the column **(A, B)** but for SNR = 5 dB and SNR = 10 dB, respectively.

In Figure 10, the AE and the number of untracked centers of the innervation zone *N*_*ne*_ are shown for the optimal parameters in the case of α = 0.5 (black crosses in Figure 8B) starting from SNR = 0 dB in column (A, D) of Figure 10. The same is shown in columns (B, E) and (C, F) for SNR = 5 dB and SNR = 10dB, respectively. As shown in Figure 10, all mean errors MAE are below the inter electrode distance (IED). With a high signal-to-noise ratio [column (C, F)], again significantly fewer motor units are tracked (small number of outliers above the IED line).

In summary, the error score ES(α) can be used to configure the presented algorithm for different use cases by adjusting α, as there might be scenarios where the accuracy could be less important compared to the robustness of the algorithm and vice versa.

## 4. Discussion

This study aimed to develop an algorithm for the localization of motor unit innervation centers in skeletal muscles based on sEMG measurements. The algorithm is intended to improve the prediction quality of muscle-driven joint models by estimating the mechanical shortening of a muscle during contraction more accurately based on the measurements of displacements of the innervation center. This information can be used, e.g., in the respective muscle submodel. In the next step, it has to be tested whether the displacement tracking of motor unit innervation centers allows for a direct determination of muscle shortening, e.g., by determining movement of a mean innervation center.

For future use in embedded systems of exoskeletons and wearables, the algorithm presented in this study has to meet several requirements. It has to be compatible with noisy measurement data and be computationally efficient at the same time. In order to objectively evaluate both requirements, it was decided to test the algorithm in this study on noisy simulated data in a controlled manner instead of directly using real data. It was shown that the algorithm can cope with artificially noisy data. For the subsequent adaptation to real data, the ability to balance the robustness of the recognition against the recognition accuracy under different noise conditions plays an important role.

During the design of the myoelectric simulator used in this study, the following key assumptions were made. The muscle fibers are assumed to be placed in a homogenous, isotropic, solely resistive, and unbound medium. The muscle fiber transmembrane currents are modeled as concentrated current sources and sinks, whose strengths and positions are functions of time. The muscle fibers themselves are of configurable length and position and have a configurable innervation point position. These assumptions allow a simulation of the end effect of the muscle fiber and gradual emergence of the signal at the innervation point of the muscle fiber. The model thus fulfills the requirements for a simulation that is used for the investigation of motor end plate zones (Stegeman et al., 2000). Additionally, the generated EMG signal shapes of the myoelectric simulator were qualitatively compared to real recordings (Roberto Merletti, 2004).

The assumptions mentioned above allow a fast and efficient computation of the simulated EMG. Petersen and Rostalski (2019) published an open source simulator that makes use of a more complex volume conductor model and a continuous source function at the cost of an increase in computational complexity. Zhang et al. (2017) proposed an EMG model using a detailed volume conductor, based on magnetic resonance imaging, utilizing finite element electric field simulation. However, both complex models were not needed to design the presented algorithm, as the typical sEMG signal wave form was already achieved by the assumptions in the presented simulation approach.

In the next steps, the algorithm will be implemented on an embedded system and tested with real measurement data. The embedded variant would have to identify multiple innervation zone centers within an EMG recording. The presented algorithm is designed to track the center of the innervation zone of a single motor unit. As a result, the algorithm has to be extended to find all motor unit centers of the innervation zone. Therefore, for e.g., an intermediate processing step needs to be established. In the current form of the presented algorithm, such an intermediate processing step would need to segment the EMG data in time, this could be as simple as a sliding window. The hypothesis, that a simple segmentation method is sufficient, is coupled with the assumption that it is sufficient to observe a mean innervation zone movement of all observed motor unit potentials instead of identifying and tracking potentials of exactly the same motor units. A more elaborate approach might be preferable which ensures that in a given window, there is a dominant motor unit potential.

In the medium term, the relationship between mean center displacement of the innervation zone and muscle contraction movement will be investigated in more detail, and the algorithm will be expanded accordingly. Future work in this regard will be focused on relatively large muscles, like the *biceps brachii*, as in the case of larger muscles, there is less chance of interference with dominant motor unit potentials from other muscles. It is to be expected that in general, any method for tracking innervation zones performs poor for muscles that are relatively small and that are close to other muscles (e.g., forearm muscles). Finally, it will be integrated into the joint control of an actuated exoskeleton.

## Data availability statement

Additional data for this paper is available at the repository: Mechenberg (2023). Supplementary Material: A method for the estimation of a motor unit innervation zone center position evaluated with a computational sEMG model (v0.0.1). Zenodo, https://doi.org/10.5281/zenodo.8056478.

## Author contributions

MM and AS contributed to the conception, design of the study, and wrote sections of the manuscript. MM implemented and designed the proposed simulation software, the algorithm, organized the data, and wrote the first draft of the manuscript. Both authors contributed to the manuscript revision and read and approved the submitted version.
